# Subacute Bartonella endocarditis with glomerulonephritis: a diagnostic and therapeutic challenge in blood culture–negative infective endocarditis

**DOI:** 10.1099/acmi.0.001051.v3

**Published:** 2025-12-18

**Authors:** James Bae, Abisola Ipaye, Joanna Pocock, Pradeep Kaul, A Ruth M. Kappeler, Sumita Pai

**Affiliations:** 1 Royal Papworth Hospital, Papworth Rd, Trumpington, Cambridge CB2 0AY, UK; 2 Department of Physiology, Development and Neuroscience, University of Cambridge, Cambridge, UK

**Keywords:** *Bartonella henselae*, cat-scratch disease, glomerulonephritis, infective endocarditis

## Abstract

**Background.** *Bartonella* species are increasingly recognized as a significant cause of blood culture–negative infective endocarditis. Their diagnosis is often challenging, leading to delays and suboptimal treatment outcomes.

**Case report.** This report includes a concise literature review and a case involving a 77-year-old male with a history of bovine aortic valve replacement. The patient presented with lethargy, fever, unintentional weight loss and acute kidney injury. Despite repeated blood cultures and extensive diagnostic evaluations yielding negative results, the definitive diagnosis was achieved post-surgery when valve PCR identified *Bartonella* species, likely linked to cat exposure. The patient was successfully treated with an extended course of doxycycline and rifampicin, leading to clinical resolution.

**Conclusion.** This case highlights the diagnostic complexities of *Bartonella* endocarditis, including negative blood cultures, subacute clinical presentation and its ability to mimic autoimmune glomerulonephritis, leading to unnecessary immunosuppressive therapy. It underscores the need for improved diagnostic approaches and clinician awareness to identify at-risk populations, such as those with cat exposure or poor hygiene, ensuring the correct diagnostic investigation for an early antibiotic intervention.

## Data Summary

All patient confidential data are anonymized. All other data required for the review of this case report are presented in the manuscript.

## Introduction

Blood culture–negative infective endocarditis (BCNIE) is an underrecognized yet clinically significant subset of infective endocarditis (IE), with an estimated prevalence of ~9%, varying widely from 2.5% to 31% depending on geographic regions [1,3]. Common causes of BCNIE include *Coxiella burnetii* (Q fever), *Bartonella* spp., *Tropheryma whipplei*, fungi, mycobacteria and the *HACEK* group of organisms [4,5]. Among these, *Bartonella* species – especially *Bartonella henselae* and *Bartonella quintana* – have become increasingly recognized as important pathogens, collectively responsible for up to 3% of all IE or nearly a quarter of BCNIE in certain cohorts [5,8].

*Bartonella* endocarditis often presents as a subacute or indolent illness characterized by nonspecific symptoms such as fever, malaise and weight loss, before progressing to severe complications, including valvular destruction, heart failure and systemic embolization [8,10]. Renal complications, particularly rapidly progressive renal failure or crescentic glomerulonephritis, are more frequently associated with *Bartonella* endocarditis than with other pathogens causing IE [11,13]. Additional manifestations include a higher prevalence of splenomegaly compared to culture-positive endocarditis, as well as central nervous system involvement, which can lead to headaches, visual disturbances, impaired coordination, seizures and memory loss [11,14].

Despite advancements in diagnostic techniques, *Bartonella* IE remains difficult to diagnose. Blood cultures are often negative. This is further complicated by the frequent administration of empirical antibiotics prior to sample collection, which reduces culture sensitivity even further. Other diagnostic methods, including serologic and molecular approaches – such as immunofluorescence assays, enzyme immunoassays and PCR – play a crucial role in identifying *Bartonella* in cases of suspected BCNIE [7,15]. Serological titres ≥1 : 800 have traditionally been considered highly sensitive; however, recent studies indicate a sensitivity range of 60–96%, highlighting the importance of confirmatory testing with PCR or Western blot [5,15]. Multimodal approaches, including combining molecular diagnostics and serological testing, have therefore become essential for accurate diagnosis. Additionally, recent case reports underscore the value of multimodality imaging in supporting diagnosis, particularly in patients who lack classic clinical features of endocarditis [16].

*Bartonella* endocarditis often presents subacutely and can be complicated by glomerulitis, resulting in frequent delays in diagnosis or misdiagnosis. Early recognition and targeted treatment are crucial to avoid the unnecessary administration of immunosuppressive therapy, which may occur in cases mistakenly identified as vasculitis [17,19]. Enhanced diagnostic methods and increased clinician awareness are essential for screening at-risk populations, particularly individuals with risk factors such as cat exposure or poor hygiene. In this article, we provide a concise review of current diagnostic and therapeutic approaches for *Bartonella* IE, followed by a case report of prosthetic valve endocarditis caused by *Bartonella* in a 77-year-old man, further complicated by crescentic glomerulonephritis.

## Case presentation

A 77-year-old man presented to the emergency department with a 2-month history of marked lethargy, fatigue and pyrexia of unknown origin, alongside a 15 kg weight loss over the preceding 12 months. His past medical history included bovine aortic valve replacement for severe aortic regurgitation 12 years earlier, dual-chamber pacemaker placement for 2 : 1 atrioventricular block 7 months prior, recent thrombocytopenia, hypercholesterolaemia, hypothyroidism and dilated ascending aorta.

On admission, his clinical frailty score was 3. Initial laboratory investigations revealed stage II acute kidney injury with haemato-proteinuria, a C-reactive protein level of 52 mg l^−1^ and pancytopenia. Two sets of blood cultures showed no growth, and three subsequent sets remained negative.

**Fig. 1.**
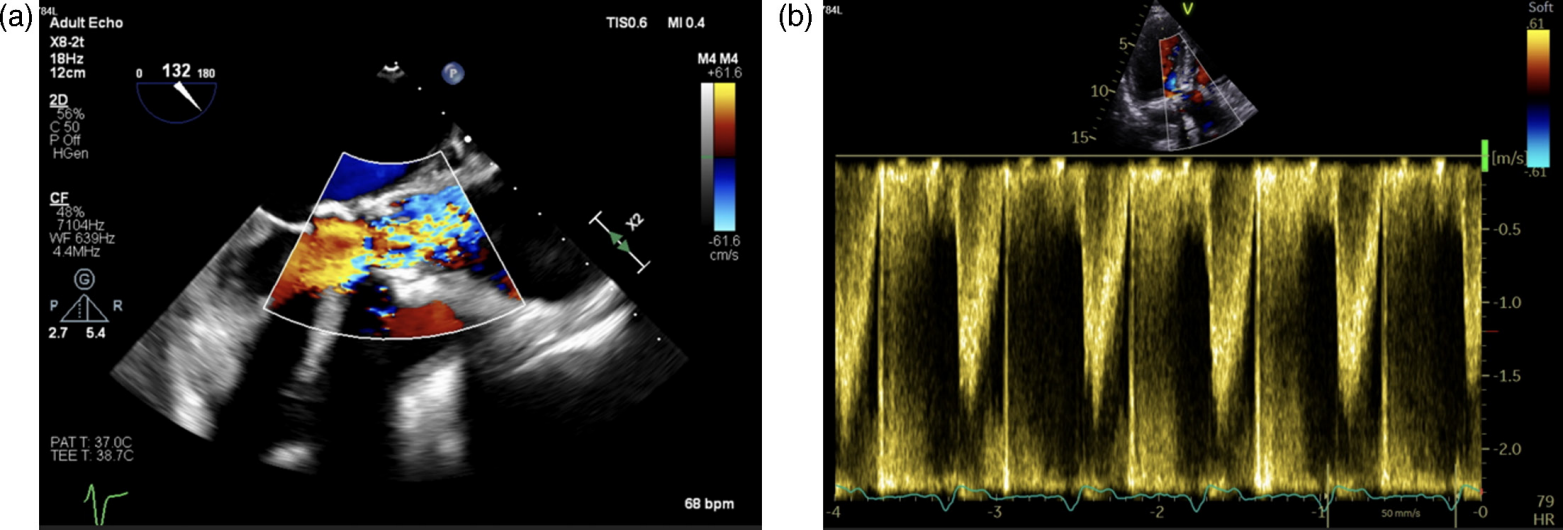
(a) Transoesophageal echocardiography shows moderate transvalvular regurgitation from a suspected tear in the right coronary cusp, with flow reversal in the descending aorta. (b) Continuous-wave Doppler confirming regurgitation with a peak velocity of 3.4 m s^−1^ and a mean gradient of 25 mmHg.

Further evaluation of his deteriorating renal function led to a renal biopsy consistent with IgA-dominant acute glomerulonephritis, suspected to be secondary to an infective aetiology (i.e. *Staphylococcus aureus or Streptococci*). Transthoracic echocardiography demonstrated degeneration of his bioprosthetic aortic valve. A subsequent transoesophageal echocardiogram (TOE) revealed new masses on the non-coronary cusp of the valve, prolapsing into the left ventricular outflow tract – changes not observed on a TOE performed 14 months earlier (Fig. 1). Positron emission tomography–computerized tomography corroborated these findings by showing evidence of infection in the prosthetic aortic valve and increased fluorodeoxyglucose uptake in thoracic lymph nodes. Renal biopsy 15 March 2023 showed IgA-dominant acute glomerulonephritis, most likely associated with infective aetiology.

Extensive investigations for culture-negative endocarditis, including testing for *Legionella*, *Aspergillus* and *Coxiella*, yielded negative results. Unfortunately, *Bartonella* serology was not performed at this time due to its low regional prevalence and the discontinuation of the assay service. Empiric antibiotic therapy initially comprised vancomycin, gentamicin and rifampicin, later modified to vancomycin plus meropenem. Ultimately, the patient underwent a redo sternotomy with valve replacement, which showed moderate to severe pericardial adhesions, but no root abscess was detected. Left ventricular function remained preserved, and the aortotomy incision was successfully reconstructed using a bovine pericardial patch. Vegetation on the valve was collected for microbiological analysis, and sequencing through nanopore technology confirmed *B. henselae*.

On further questioning, the patient later reported introducing a new cat into his household 3 years earlier, which had not been house-trained or screened for fleas. Following the diagnosis, he was treated with a 6-week course of doxycycline (100 mg twice daily) and a 4-week course of rifampicin (300 mg), resulting in the clinical resolution of the infection. The patient was followed up after 4 weeks, with no reported complications.

## Discussion

BCNIE represents a significant subset of endocarditis cases, predominantly involving intracellular organisms such as *C. burnetii* and *Bartonella* spp. [1,8, 15]. Among these pathogens, *C. burnetii* is the most reported cause of culture-negative endocarditis, followed by *Bartonella* spp., although the distribution may vary regionally [3]. Within the *Bartonella* genus, *B. henselae* and * B. quintana* account for more than 90% of endocarditis cases: *B. henselae* is associated with cat-scratch disease (domestic cats serving as the primary reservoir), whereas *B. quintana* – the causative agent of trench fever – is transmitted by the human body louse, often in settings of overcrowding and poor hygiene [8,20].

As demonstrated in this case report, most patients with *Bartonella* endocarditis present with a subacute syndrome, commonly characterized by low-grade fever, malaise, weight loss and other nonspecific systemic symptoms [5,7, 21]. The bacteria preferentially adhere to and infect the endothelium, typically affecting native or previously damaged cardiac valves [22, 23]. This chronic infection often elicits granulomatous inflammation, leading to the formation of vegetations that may cause valvular dysfunction or embolic events [24]. These vegetations are found to be very common in *Bartonella* endocarditis, ~90% of cases, with aortic valve involvement in 59–85% of patients [25,26]. Unlike endocarditis caused by more virulent organisms, such as *S. aureus*, the subacute course of *Bartonella* endocarditis can persist for weeks to months before overt clinical symptoms emerge. Additionally, its atypical presentations may mimic other conditions, leading to potential misdiagnosis and contributing to diagnostic challenges and delays in starting the appropriate treatment.

In addition to its subacute presentation and predominant valve involvement, *Bartonella* endocarditis also frequently manifests with renal complications. One of the key features is crescentic glomerulonephritis leading to renal failure, observed in ~30–50% of patients [18,27,29]. Notably, *Bartonella* endocarditis is associated with a high prevalence (70–80%) of antineutrophil cytoplasmic antibody (ANCA). For instance, one review identified 54 cases of *Bartonella*-induced IE complicated by glomerulonephritis, of which 77% were serologically positive for ANCA [19]. Similarly, in a series of 24 patients with *Bartonella* infection, 83% tested positive for ANCA-PR3, and 92% developed glomerulonephritis – rates significantly higher than the ~18% renal complication rate observed in general IE cases [30,31]. Because ANCA-PR3 is commonly associated with rapidly progressive glomerulonephritis, the use of corticosteroids is often initiated, potentially delaying definitive diagnosis and management. As illustrated in our case, the secondary glomerulonephritis associated with *Bartonella* endocarditis can mimic vasculitis, complicating the diagnostic process and highlighting the need for early recognition and targeted management.

The guideline for diagnosis of *Bartonella* endocarditis is currently not established and often requires multiple modalities to confirm the diagnosis, including serology, molecular assays and histopathological evaluation. Indirect fluorescent antibody (IFA) serology is typically the most accessible initial method, enabling the detection of elevated IgG titres. Although recent evidence indicates that the conventional threshold (*Bartonella*-specific IgG titre ≥1 : 800) may yield a sensitivity as low as 60% in some patient populations, this titre remains a principal benchmark for diagnosing *Bartonella* infections [6,15]. In the UK, serological testing has been largely withdrawn due to its limited sensitivity and potential cross-reactivity with *Coxiella* serology. However, some centres (e.g. RIPL) still offer serological tests when there is a high clinical suspicion.

Additional modalities, including reverse transcription PCR and Western blot, can further enhance diagnostic accuracy. Notably, 16S rRNA amplification from blood specimens may have a sensitivity range of 13.6–30%, whereas molecular testing performed on excised valvular tissue and Western blots on blood can reach up to 92% and 100%, respectively [5,31]. One study found that the presence of any one of the following, a positive PCR result from blood, an IFA titre ≥1 : 800 and a positive Western blot, provided 100% sensitivity for detecting *Bartonella* in 102 patients, proposing these metrics as potential major criteria for diagnosing *Bartonella* endocarditis in blood culture–negative endocarditis [5].

Other diagnostic modalities, particularly transoesophageal echocardiography, remain essential for visualizing vegetations and confirming structural abnormalities. In cases where echocardiographic findings are inconclusive, cardiac magnetic resonance imaging can provide valuable supplementary information [32]. Additionally, in patients with concurrent renal involvement, a renal biopsy may be warranted, as distinct histopathological patterns, such as immune complex deposition and necrotizing glomerular lesions, can point to infection-related pathology and thus aid in diagnosis [11,33].

The treatment of *Bartonella* endocarditis requires prolonged courses of antimicrobial agents with intracellular activity. Management is further complicated by the likely causative pathogen and the presence of prosthetic material. Additionally, current treatment guidelines for endocarditis exhibit significant variability (Table 1).

**Table 1.** Summary of reported antimicrobial regimens for the treatment of bartonella endocarditis [26,34,40]

Paper	Guidance	Comments
Raoult *et al*. 2003 [26]	2-week course of **gentamicin**, in combination with a prolonged duration of **β-lactam** or **doxycycline**	The recovery rate was significantly higher, and prognosis improved with a 2-week aminoglycoside combination therapy (*P*=0.02). The least effective regimens were those containing doxycycline without aminoglycosides.
Rolain *et al*. 2004 [34]	**Gentamicin** is given for the initial 2 weeks, followed by **doxycycline** administration for 6 weeks.	Review on Antimicrobials for *Bartonella* spp. Gentamicin, among aminoglycosides, showed the lowest MIC against *Bartonella* spp. Macrolides demonstrated high efficacy, while cephalosporins were moderately effective.
BSAC 2012 [35]	**Gentamicin** in combination with a **b-lactam** or **doxycycline** for a minimum of 4 weeks.	Guideline Only aminoglycosides have bactericidal activity against Bartonella spp., although susceptibility to macrolides, rifampicin and tetracycline has been demonstrated.
Ghashghaei *et al*. 2015 [36]	2 weeks of **gentamicin** plus 6 weeks of **ceftriaxone** with or without **doxycycline**	A case report Treatment recommendations for *Bartonella* endocarditis are based on case series.
Papineni *et al*. 2017 [37]	2 weeks with **gentamicin** and 3 months with **doxycycline**	Two case reports of *B*. *henselae* Treated without cardiac vulvectomy
Noopetch *et al*. 2018 [38]	Varying regimens Patients were treated with **ampicillin**, **gentamicin**, **ceftriaxone**, **levofloxacin**, **doxycycline** and **azithromycin**	7 case series reports and literature reviews
Dietz *et al*. 2021 [39]	**Gentamicin** 1 mg kg^−1^ tds first 2 weeks+**doxycycline** 100 mg IV/PO×6 weeks	Case report and literature review Antibiotics with activity: macrolides/doxycycline In combination: Rif/gent/ceftriaxone Drugs that are generally not active: Cipro/TMP-SMX, most cephalosporins/penicillins/aztreonam If valve resected: Doxy for another 6 weeks If no valve surgery: Doxy for another 3 months
ESC 2023 [40]	**Gentamicin** (3 mg/24 h) i.v. for 2 weeks followed by **doxycycline** 100 mg/12 h orally for 4 weeks	Other treatments: ampicillin or amoxicillin (12 g/24 h i.v.) or cephalosporins (ceftriaxone 2 g/24 h i.v.) combined with aminoglycosides (gentamicin or netilmicin).

Based on various studies, a combination therapy of doxycycline and an aminoglycoside (e.g. gentamicin) for at least 2 weeks, followed by an extended course of oral doxycycline, is commonly recommended to achieve optimal clinical outcomes [26,34,40]. However, the use of aminoglycosides carries significant risks, including nephrotoxicity and ototoxicity, particularly in *Bartonella* endocarditis, where renal impairment is a common manifestation [41,42]. Therefore, renal function and therapeutic drug monitoring are essential. In cases where aminoglycosides are not feasible, a combination of rifampicin and doxycycline has been suggested as an alternative.

The duration of treatment varies depending on surgical intervention. For example, 6 weeks of doxycycline alone has been shown to be sufficient when valvectomy is performed, while a total of 3 months of doxycycline is recommended for conservatively managed cases [39,40]. Despite the increasing recognition of *Bartonella* endocarditis, the optimal treatment regimen remains undefined. This highlights the need for further trials to establish evidence-based antimicrobial strategies.

## Conclusion

*Bartonella* endocarditis presents significant challenges in diagnosis and management due to its subacute nature, atypical clinical features and associated renal complications. This case underscores the insidious progression of the disease and the complexities of its presentation, including IgA glomerulonephritis and persistently negative blood cultures. Recognizing the condition requires careful consideration of clinical and environmental factors, particularly in patients with prosthetic valves or cat exposures. Comprehensive diagnostic strategies, including serological assays, PCR testing and valve tissue cultures, are often critical to identifying the causative organism. These challenges underscore the importance of developing standardized diagnostic criteria, enhancing clinician awareness and establishing evidence-based treatment protocols to facilitate effective management of *Bartonella* endocarditis.

## Abbreviations

ANCA: antineutrophil cytoplasmic antibody
BCNIE: blood culture–negative infective endocarditis
IFA: indirect fluorescent antibody
TOE: transoesophageal echocardiogram

## References

[1] Godfrey R, Curtis S, Schilling WH, James PR (2020). Blood culture negative endocarditis in the modern era of 16S rRNA sequencing. Clin Med (Lond).

[2] Van Scoy RE (1982). Culture-negative endocarditis. Mayo Clin Proc.

[3] Tattevin P, Watt G, Revest M, Arvieux C, Fournier PE (2015). Update on blood culture-negative endocarditis. Med Mal Infect.

[4] Brouqui P, Raoult D (2001). Endocarditis due to rare and fastidious bacteria. Clin Microbiol Rev.

[5] Edouard S, Nabet C, Lepidi H, Fournier PE, Raoult D (2015). Bartonella, a common cause of endocarditis: a report on 106 cases and review. J Clin Microbiol.

[6] Raoult D, Fournier PE, Drancourt M, Marrie TJ, Etienne J (1996). Diagnosis of 22 new cases of *Bartonella endocarditis* [published correction appears in Ann Intern Med 1997 Aug 1;127(3):249]. Ann Intern Med.

[7] Houpikian P, Raoult D (2005). Blood culture-negative endocarditis in a reference center: etiologic diagnosis of 348 cases. Medicine (Baltimore).

[8] Okaro U, Addisu A, Casanas B, Anderson B (2017). *Bartonella* species, an emerging cause of blood-culture-negative endocarditis. Clin Microbiol Rev.

[9] Edouard S, Nabet C, Lepidi H, Fournier PE, Raoult D (2021). Bartonella, a cause of blood-culture-negative endocarditis. Diagn Microbiol Infect Dis.

[10] Hill EE, Herijgers P, Herregods M-C, Peetermans WE (2006). Evolving trends in infective endocarditis. Clin Microbiol Infect.

[11] Andeen NK, Kung VL, Nguyen JK, Avasare RS, Nakhoul GN (2025). *Bartonella* endocarditis-associated glomerulonephritis: a mimicker of autoimmunity and vasculitis. Kidney Int Rep.

[12] Shrestha NK, Kanyo EC, Nakhoul GN, Herlitz LC, Gordon SM (2024). Association between causative pathogen and occurrence of infection-related glomerulonephritis in infective endocarditis. Clin Infect Dis.

[13] Babiker A, El Hag MI, Perez C (2018). Bartonella infectious endocarditis associated with cryoglobulinemia and multifocal proliferative glomerulonephritis. Open Forum Infect Dis.

[14] Breitschwerdt EB, Maggi RG, Nicholson WL, Cherry NA, Woods CW (2008). *Bartonella sp*. bacteremia in patients with neurological and neurocognitive dysfunction. J Clin Microbiol.

[15] Fournier P-E, Mainardi J-L, Raoult D (2002). Value of microimmunofluorescence for diagnosis and follow-up of *Bartonella* endocarditis. Clin Vaccine Immunol.

[16] Vinod P, Khayata M, Haouzi A, Xu B (2025). Role of multimodality imaging in infective endocarditis: a comparison of the major society guidelines in the United States and Europe. Trends Cardiovasc Med.

[17] Bannon L, Choshen G, Giladi M, Ablin J (2019). B artonella endocarditis masquerading as systemic vasculitis with rapidly progressive glomerulonephritis (aka ‘löhlein nephritis’). BMJ Case Report.

[18] Vercellone J, Cohen L, Mansuri S, Zhang PL, Kellerman PS (2018). *Bartonella* endocarditis mimicking crescentic glomerulonephritis with PR3-ANCA positivity. Case Rep Nephrol.

[19] Beydon M, Rodriguez C, Karras A, Cez A, Rafat C (2022). *Bartonella* and coxiella infections presenting as systemic vasculitis: case series and review of literature. Rheumatology (Oxford).

[20] Fournier PE, Lelievre H, Eykyn SJ, Mainardi JL, Marrie TJ (2001). Epidemiologic and clinical characteristics of *Bartonella quintana* and *Bartonella henselae* endocarditis: a study of 48 patients. Medicine (Baltimore).

[21] Lamas CC, Eykyn SJ (2003). Blood culture negative endocarditis: analysis of 63 cases presenting over 25 years. Heart.

[22] Meidrops K, Groma V, Goldins NR, Apine L, Skuja S (2023). Understanding *Bartonella*-Associated Infective endocarditis: examining heart valve and vegetation appearance and the role of neutrophilic leukocytes. Cells.

[23] James EA, Hill J, Uppal R, Prentice MB (2000). *Bartonella* infection: a significant cause of native valve endocarditis necessitating surgical management. J Thorac Cardiovasc Surg.

[24] Lepidi H, Fournier PE, Raoult D (2000). Quantitative analysis of valvular lesions during *Bartonella* endocarditis. Am J Clin Pathol.

[25] García-Álvarez L, García-García C, Muñoz P, Fariñas-Álvarez MDC, Cuadra MG (2022). *Bartonella* endocarditis in Spain: case reports of 21 cases. Pathogens.

[26] Raoult D, Fournier P-E, Vandenesch F, Mainardi J-L, Eykyn SJ (2003). Outcome and treatment of *Bartonella endocarditis*. Arch Intern Med.

[27] Raybould JE, Raybould AL, Morales MK (2016). *Bartonella endocarditis* and pauci-immune glomerulonephritis: a case report and review of the literature. Infect Dis Clin Pract.

[28] Zhang W, Zhang H, Wu D, Fu H, Shi W (2020). Antineutrophil cytoplasmic antibody-positive infective endocarditis complicated by acute kidney injury: a case report and literature review. J Int Med Res.

[29] Boils CL, Nasr SH, Walker PD, Couser WG, Larsen CP (2015). Update on endocarditis-associated glomerulonephritis. Kidney Int.

[30] Habib G, Erba PA, Iung B, Donal E, Cosyns B (2019). Clinical presentation, aetiology and outcome of infective endocarditis. Results of the ESC-EORP EURO-ENDO (European infective endocarditis) registry: a prospective cohort study. Eur Heart J.

[31] Fournier P-E, Thuny F, Richet H, Lepidi H, Casalta J-P (2010). Comprehensive diagnostic strategy for blood culture-negative endocarditis: a prospective study of 819 new cases. Clin Infect Dis.

[32] Bruun NE, Habib G, Thuny F, Sogaard P (2014). Cardiac imaging in infectious endocarditis. Eur Heart J.

[33] Yoshifuji A, Hibino Y, Komatsu M, Yasuda S, Hosoya K (2021). Glomerulonephritis caused by *Bartonella spp*. infective endocarditis: the difficulty and importance of differentiation from anti-neutrophil cytoplasmic antibody-related rapidly progressive glomerulonephritis. Intern Med.

[34] Rolain JM, Brouqui P, Koehler JE, Maguina C, Dolan MJ (2004). Recommendations for treatment of human infections caused by *Bartonella* species. Antimicrob Agents Chemother.

[35] Gould FK, Denning DW, Elliott TSJ, Foweraker J, Perry JD (2012). Guidelines for the diagnosis and antibiotic treatment of endocarditis in adults: a report of the working party of the british society for antimicrobial chemotherapy. J Antimicrob Chemother.

[36] Ghashghaei R, Thung I, Lin GY, Sell RE (2016). Bartonella endocarditis. J Cardiol Cases.

[37] Papineni P, Carroll A, Radvan J, Hemsley C, Chambers J (2017). Management of *Bartonella* prosthetic valve endocarditis without cardiac surgery. *Emerg Infect Dis*.

[38] Noopetch P, Ponpinit T, Suankratay C (2018). *Bartonella henselae* infective endocarditis with dissemination: a case report and literature review in Southeast Asia. IDCases.

[39] Dietz BW, Winston LG, Koehler JE, Margaretten M (2021). Copycat. N Engl J Med.

[40] Delgado V, Ajmone Marsan N, de Waha S, ESC Scientific Document Group (2023). ESC guidelines for the management of endocarditis: developed by the task force on the management of endocarditis of the european society of cardiology (ESC). Eur Heart J.

[41] Le TA, Hiba T, Chaudhari D, Preston AN, Palowsky ZR (2023). Aminoglycoside-related nephrotoxicity and ototoxicity in clinical practice: a review of pathophysiological mechanism and treatment options. Adv Ther.

[42] Croes S, Koop AH, van Gils SA, Neef C (2012). Efficacy, nephrotoxicity and ototoxicity of aminoglycosides, mathematically modelled for modelling-supported therapeutic drug monitoring. Eur J Pharm Sci.

